# Primary orbital rhabdomyosarcoma with skeletal muscle metastasis

**DOI:** 10.4103/0974-620X.64235

**Published:** 2010

**Authors:** Jayanta K. Das, B. K. Tiwary, S. B. Paul, Harsha Bhattacharjee, Bhuyan Cida, Dipankar Das

**Affiliations:** Department of Ophthalmology, Sri Sankaradeva Nethralaya, Guwahati, India; 1 Department of Life Science, Assam University Silchar, Medical Oncology, Guwahati, India; 2 Department of Chemistry, B.B Cancer Institute, Guwahati, India

**Keywords:** Chemotherapy and radiotherapy, exenteration, rhabdomyosarcoma

## Abstract

We report a case of embryonal orbital rhabdomyosarcoma (RMS) in a five year old boy. Immuno-histochemistry of incisional specimen confirmed diagnosis. Eight cycles of chemotherapy along with radiotherapy resulted in over 50% reduction in size of the mass. However, increase in size was noted subsequent to completion of therapy and exenteration was deemed prudent. Margins of the excised specimen were free from tumor cells, but after five months, the patient developed multiple metastases, including skeletal muscle involvement, and died nine months after exenteration, despite repeat chemotherapy along with radiotherapy. Orbital RMS with metastasis to skeletal muscle is a rare entity.

## Introduction

Rhabdomyosarcoma (RMS) is the most common primary malignancy of the orbit in children, accounting for 4% of all malignant disease in children.[1,2] Horn and Enterline in mid 1900 first classified RMS histologically into 4 major categories: embryonal, alveolar, botryoid embryonal and pleomorphic, which was later supported by other reserchers.[2] Prognostically pleomorphic variety has best prognosis.[2] The average age of presentation is 4-7 years.[3] Most of the time the tumor is retro-bulbar, but it may arise from any part of the orbit, even from the conjunctiva and anterior uveal tract.[4] The majority of tumors are localized to the orbit. In its highly malignant form, RMS grows rapidly and behaves aggressively, frequently invading adjacent bones and soft tissues. Extension of tumor outside the orbit is associated with worse prognosis particularly if there is skull base erosion. Though earlier orbital RMS was treated by exenteration, in 1979, Abraham *et al*. demonstrated irradiation alone or in combination with chemotherapy to be more effective than exenteration for both control and long term survival. Marked improvement in RMS survival over the past seen with the use of recent therapeutic protocols including primary radiotherapy and chemotherapy.[5]

## Case Report

**Figure 1:**
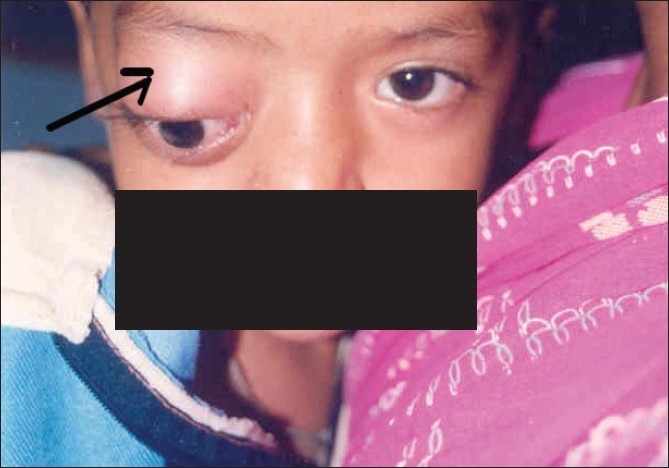
Clinical photograph of the patient showing downward eccentric protrusion of right eyeball (arrow)

**Figure 2:**
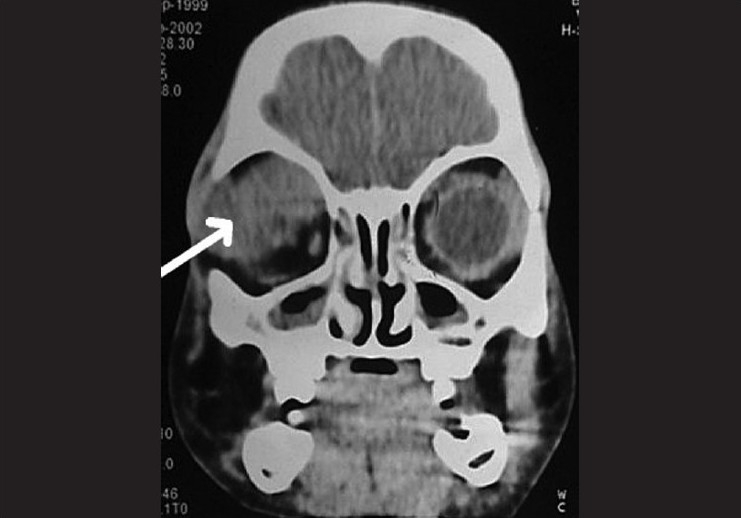
Coronal CT scan of the orbits showing a superior right orbital mass (arrow) displacing the globe inferiorly

**Figure 3:**
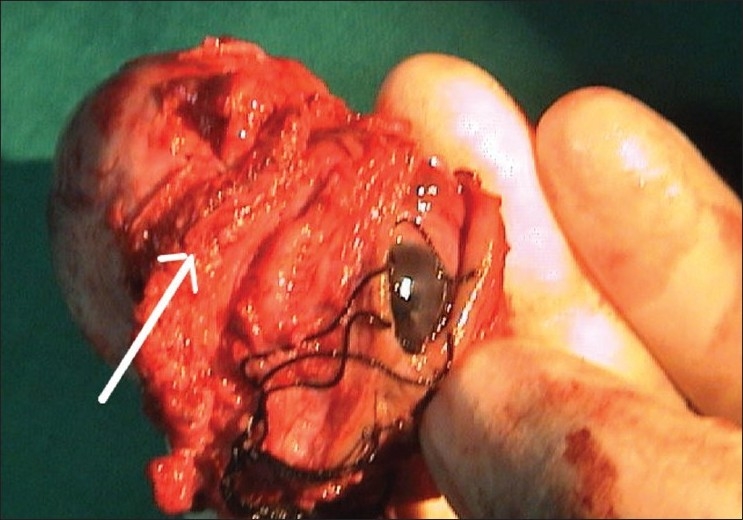
Exenterated gross specimen (arrow)

**Figure 4:**
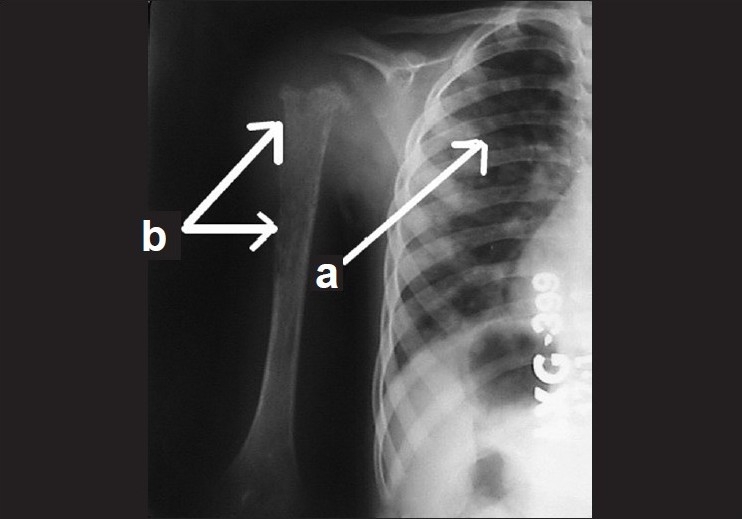
X-ray of the chest (a) and right shoulder joint (b) showing metastatic changes

**Figure 5:**
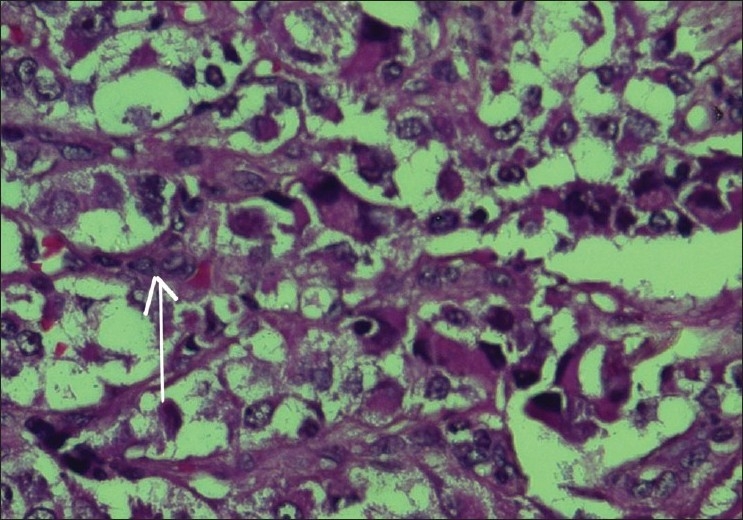
Histological view showing spindle cell proliferation arranged in a palisading fashion, exhibiting small hyperchromatic nuclei (arrow) suggestive of embryonal cell rhabdoomyosarcoma

A five-year-old boy presented to us with rapid onset of downward eccentric protrusion of right eyeball over a period of one month. [Figure 1] Extra ocular muscle movements were grossly restricted in all direction. He had no other medical problem. On examination, his visual acuity was 20/100 OD, which was improved to 20/25 by refractive correction (-0.75,-2.5x180°) and 20/20 (OS). Exophthalmometry revealed 23mm and 16mm in right and left side respectively. Computerized tomography (CT) scan revealed a retro bulbar superior orbital soft tissue mass compressing the optic nerve and displacing the globe inferiorly [Figure 2]. Regional lymph nodes were not palpable and there was no distant metastasis observed on detailed work-up which include complete blood examination, liver function test, chest X-ray, ultrasonography of whole abdomen and bone marrow aspiration. Incisional biopsy was performed and histopathological examination of the biopsy specimen revealed embryonal RMS. The histopathological diagnosis was confirmed by immuno-histochemical study which showed positive reaction to anti- myoglobin and anti-desmine antibodies. The child was put on eight cycles of cisplatin based chemotherapy (bleomycin-etoposide-cisplatin) along with radiotherapy under the supervision of medical oncologist and radiologist, and this resulted in over 50% reduction in size of the mass. However, increase in size subsequent to completion of therapy resulted in a significant residual mass. A detailed work up to exclude distant metastasis was repeated at this stage with no positive findings noted. Considering the increasing size of the residual mass and the absence of distant metastases, exenteration was carried out in consultation with the medical oncologist and radiologist. Margins of the excised specimen were free from tumor tissue [Figure 3]. However, five months after exenteration, the patient developed multiple metastases, including skeletal muscle (deltoid of the right shoulder joint) involvement [Figure 4]. Incisional biopsy specimen of deltoid muscle confirmed metastatic RMS [Figure 5]. Another course of chemotherapy along with radiotherapy was administered; the patient however died nine months after exenteration.

## Discussion

The orbital RMS is usually fast growing an infiltrative and often appears as an enlarging, painless mass. The initial symptoms may mimic differential diagnosis which includes inflammetry process, orbital cellulitis, metastatic neuroblastoma, chloroma, lymphangioma and ruptured dermoid cyst.

In orbital RMS metastases are commonly hematogenous in origin; spread most often occurs to the lungs, liver and bones.[5] Regional lymph node metastases are rare except in advanced disease, because the posterior orbit is relatively devoid of lymphatic tissue.[3] Orbital RMS is less likely to metastasize compared to RMS arising in other sites.[3] Recurrence or metastatic spread of RMS usually occurs within three years of treatment,[6] as was observed in our case.

Intergroup Rhabdomyosarcoma Study Group (IRSG) whose protocols now from the backbone of modern treatment of rhabdomyosarcoma.[7] The prognosis of children with rhabdomyosarcoma is determined by clinical group, stage, histology and age at presentation. The four clinical stages are based on tumor respectability group I, complete resection, group II, gross resection with microscopic residual tumor or complete resection with involved nodes, extension into adjacent organs or both; group III, gross residual disease; and group IV, distant metastasis at diagnosis.[7] As per IRSG staging our case was in stage III.

Recently excision biopsy is preferred more than incisional biopsy as it confirms diagnosis as well as it removes the tumor too, which is significantly helpful for better outcome.[7] Exenteration is indicated in cases of incomplete tumor regression or in cases of recurrence after treatment with chemotherapy and radiotherapy. Embyonal RMS is relatively refractory to chemotherapy.[8] The exenteration procedure, specially for refractory RMS is valuable in prolonging the survival rate.[6]

Though tumor-free margins are thought to be a key element in establishing surgical cure of a patient with any form of neoplasia, which was the case following exenteration in our patient, few published reports,[9,10] highlight that tumor-free margins have little effect on long time survival. The clear margins may prevent local recurrence, but they do not prevent micro-metastasis to lymph nodes or distant organs via the blood stream. The IRSG-V protocols are risk-based and refine to identify other factors that may distinguish patients with favorable features from those who need more intensive therapy. A new protocol that takes into account their previous treatment is needed for patients with recurrent disease.[11] However distant metastasis to skeletal muscle and corresponding long bone, especially in orbital RMS, as was seen in our patient is very rare.
